# Study on axial compressive behavior and mesoscopic structural evolution of FRP confined coal gangue concrete

**DOI:** 10.1371/journal.pone.0322647

**Published:** 2025-05-09

**Authors:** Qingwen Li, Mengjiao Xu, Chuangchuang Pan, Lei Zhang, Yuqi Zhong, Wenxia Li, Shuhan Gu, Mengmeng Yu

**Affiliations:** 1 School of Civil and Architectural Engineering, Liaoning University of Technology, Jinzhou, China; 2 China Building Materials Industry Geologic Exploration Center Liaoning Branch, Shenyang, China; 3 College of Water Resources and Hydropower Engineering, North China Electric Power University, Beijing, China; University of Sharjah, UNITED ARAB EMIRATES

## Abstract

To address the environmental hazards caused by coal gangue waste, coal gangue concrete (CGC) has been proposed as a solution. However, due to its porosity and low strength, CGC faces numerous challenges in practical applications. To further improve its performance, fiber reinforced polymer (FRP) material was introduced to confine it. In this study, the PFC3D-FLAC3D coupling analysis method was employed to simulate the uniaxial compression test of FRP confined coal gangue concrete specimens. The influence of different FRP types (GFRP, CFRP, BFRP) and coal gangue replacement rates (0%, 50%, 100%) on the axial compression performance of coal gangue concrete columns was analyzed. Based on the indoor uniaxial compression test of glass fiber reinforced polymer (GFRP) confined coal gangue concrete, the modeling and calibration of coal gangue concrete columns confined by different FRP sheets were conducted. The strength variation and microstructure evolution mechanism of coal gangue concrete specimens confined by three kinds of FRP were discussed. The results indicated that the numerical model is highly accurate and consistent with existing experiments. The type of FRP significantly influences the confinement effect on coal gangue concrete specimens. As the coal gangue replacement rate increases, both the strength and elastic modulus of the specimens decrease. The difference of the spatial distribution of strong contact number and strong contact force reflect the microscopic manifestation of the macroscopic strength. The crack evolution of FRP confined coal gangue concrete went through three stages during uniaxial compression. This study is of great significance for selecting the appropriate type of FRP confinement for concrete under different coal gangue replacement rates.

## 1 Introduction

With the rapid development of global economy, the exploitation of coal in China has been steadily increasing, resulting in a sharp rise in the amount of coal gangue produced in mines. Coal gangue is the primary solid waste generated during the coal mining process. Coal gangue is often deposited in the open air around the mine, which not only causes serious pollution and degradation of air, land, water resources and ecological system, but also causes geological disasters such as soil erosion, landslide and debris flow, which endangers human health [1–2]. Therefore, in response to the call for a green economy, the resource utilization of coal gangue is an urgent task.

In recent years, coal gangue is often used to replace coarse or fine aggregate in concrete production. The research showed that different coal gangue replacement rates have significant effects on the mechanical properties of concrete. Among them, Wang et al. [3] carried out a series of tests on short columns of concrete-filled steel tubes with varying replacement rates of spontaneously combusted coal gangue aggregate. The results indicated that as the replacement rate of spontaneously combusted coal gangue aggregate increased, the load-bearing capacity of the concrete filled steel tube short column significantly decreased, while the corresponding strain increased. Zhang et al. [4] found that the compressive strength of concrete with 100% replacement rate of spontaneously combusted coal gangue aggregate was 15.3%, 16.7%, 22.0%, and 21.6% lower than that of concrete with natural aggregates at 7, 14, 28, and 90 days, respectively. In addition to compressive strength, Wang et al. [5] also found that compared with natural aggregate concrete, the 28-day elastic modulus of coarse coal gangue concrete decreased by 23% and 32%, while the 360-day drying shrinkage rate increased by 62% to 92%. In summary, using coal gangue as a substitute for coarse aggregates in concrete not only effectively reduces coal gangue accumulation and mitigates its potential environmental impact, but also decreases the demand for natural aggregates, thereby protecting natural resources and promoting ecological balance. Furthermore, since the cost of coal gangue is generally lower than that of natural aggregates, it can effectively reduce the production cost of concrete when used as an alternative material. From the perspective of sustainable development, the application of coal gangue in concrete aligns with the principles of circular economy, realizing the reuse of waste materials while reducing resource waste and environmental pressure. This approach not only contributes to the creation of resource-efficient and environment-friendly society, but also plays a crucial role in promoting the goals of sustainable development. However, the application of coal gangue to infrastructure construction can address the issue of solid waste treatment of coal gangue, but the coal gangue concrete has the disadvantages of low strength, low stiffness and high brittleness, which limits its application in practical projects.

FRP is widely used for the reinforcement of coal [6–9], rock [10], concrete [11–15] and other structures due to its superior properties. The research showed that the strength of CGC can be significantly improved by using FRP sheet as external confinement, and the brittle failure of CGC can be avoided effectively. In recent years, researchers have conducted numerous studies on the compressive performance of FRP confined concrete, considering factors such as the FRP thickness [16,21], the coal gangue replacement rate [22], the strength of concrete [16–23] and the size of the component [24]. Zhao et al. [25] proposed that FRP confinement can reduce the harmful effects of coal gangue concrete. To improve the low strength and brittleness of coal rejects concrete, Ren T et al. [16] proposed the structural form of FRP confined coal rejects concrete column. The results showed that when the coal rejects concrete with low strength is confined, it demonstrates a significantly enhanced axial deformation capacity. Shi et al. [26] demonstrated that FRP wrapping significantly improved the nominal tensile strength of cement mortar-coal composite disk and reduced the anisotropy of the sample. In recent years, most studies have analyzed the axial compressive properties of concrete confined by carbon fiber reinforced polymer (CFRP) and GFRP. For CFRP confined concrete, Guan et al. [27] conducted a monotonic axial loading test on CFRP confined cylindrical concrete specimens. The results indicated that the compressive strength and ultimate axial strain of CFRP confined concrete were 1.29 to 1.92 times and 5.27 to 9.29 times higher than those of unconfined concrete, respectively. Wang et al. [28] investigated the mechanical properties of CFRP confined gangue aggregate concrete columns and found that CFRP confinement significantly enhanced the compressive strength and ductility of the gangue aggregate concrete. Pan et al. [29] conducted axial compression tests on concrete with circular and square cross-sections respectively. The results showed that for concrete columns with circular section, the reduction of peak stress and strain increases as the number of CFRP layers increased, while for square section columns, the decrease of peak stress is proportional to the number of CFRP layers. For GFRP confined concrete, Guan et al. [30] conducted axial monotonic loading tests on 36 spontaneous combustion gangue concrete samples. The results showed that GFRP confinement significantly enhanced both the strength and deformation capacity of the concrete. Zhang et al. [31] pointed out that GFRP confinement significantly improved the mechanical properties of coral aggregate concrete, improving the strength of the composite columns and reducing the brittleness of the coral aggregate concrete. In summary, most existing studies focus on the axial compression properties of concrete under single FRP confinement, such as CFRP or GFRP, with few studies on basalt fiber reinforced polymer (BFRP) with lower cost. However, the axial compressive properties of concrete may vary greatly under different types of FRP confinement. In addition, systematic studies on the use of coal gangue as partial or complete replacement for coarse aggregate in concrete under FRP confinement are relatively scarce. Therefore, to fill the research gap, it is necessary to understand the influence of different FRP types on the axial compressive properties of concrete.

At present, the study of mechanical properties of FRP confined coal gangue concrete primarily relies on laboratory axial compression test. However, laboratory tests can only reflect the macroscopic mechanical behavior of materials, making it difficult to reveal the underlying mechanisms of microstructural evolution. The discrete element method (DEM) based on Newton’s law of motion can reveal the micromechanical properties of concrete [32–33]. However, using only the basic unit ball in PFC to simulate laboratory FRP confined concrete samples can substantially reduce computational efficiency. In recent years, the coupling method of finite different method (FDM) and discrete element method (DEM) has attracted the attention of researchers both domestically and internationally. Wu et al. [17] employed FDM-DEM coupling method to conduct numerical axial compression tests and analysis on FRP confined concrete. The results showed that the thicker the FRP jacket, the greater the compressive strength of concrete. Jing et al. [34] simulated the indoor triaxial test of soybean particle materials by using the discrete-continuous (PFC-FLAC) coupling method. The research showed that the PFC-FLAC coupling method effectively described the macroscopic stress-strain relationship, deformation and damage characteristics, as well as the shear strength mechanical properties of soybean particle materials.

**Fig 1:**
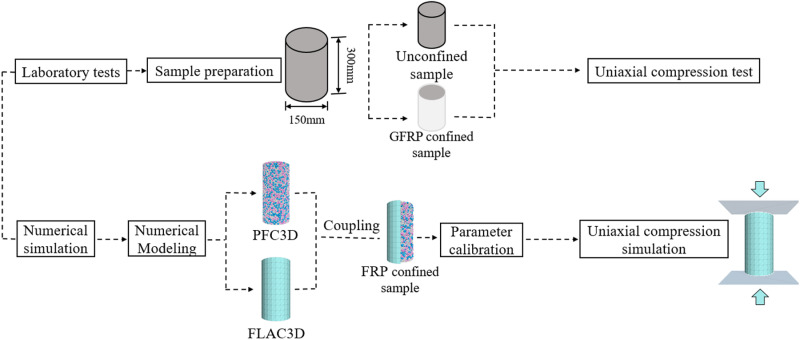
The schematic of the mechanism of action.

Although the influence of FRP on the axial compression performance of CGC has been deeply discussed in existing researches, most studies have focused on single type of FRP confinement (such as CFRP or GFRP), with limited investigation into the differences in axial compression performance among various types of FRP. Additionally, current research primarily relies on laboratory tests, with few studies employing the PFC-FLAC coupled analysis method to explore the microstructural evolution mechanisms of FRP confined CGC. Although traditional experimental studies can provide reliable data for revealing the dominant mechanism of engineering problems, they are time-consuming and costly [35]. Therefore, there is a lack of an effective simulation method to fully understand the mechanical properties of different types of FRP confined CGC under different replacement rates, especially in terms of the evolution of the microstructure. Therefore, this study uses PFC3D-FLAC3D coupled method to simulate the axial compression behavior of FRP confined CGC specimens based on laboratory uniaxial compression tests, with partial or complete replacement of coarse aggregates by coal gangue, the mechanism of action is illustrated in Fig 1. The effects of FRP type and coal gangue replacement rate on the stress-strain behavior, compressive strength, elastic modulus, strong contact number, strong contact force and crack propagation characteristics of concrete were analyzed from both macro and micro perspectives. The effect of different FRP types on the performance improvement of CGC was revealed, providing a theoretical basis and research methods for selecting the appropriate FRP type and promoting the resource utilization of coal gangue.

## 2 Laboratory tests

### 2.1 Materials

**Fig 2:**
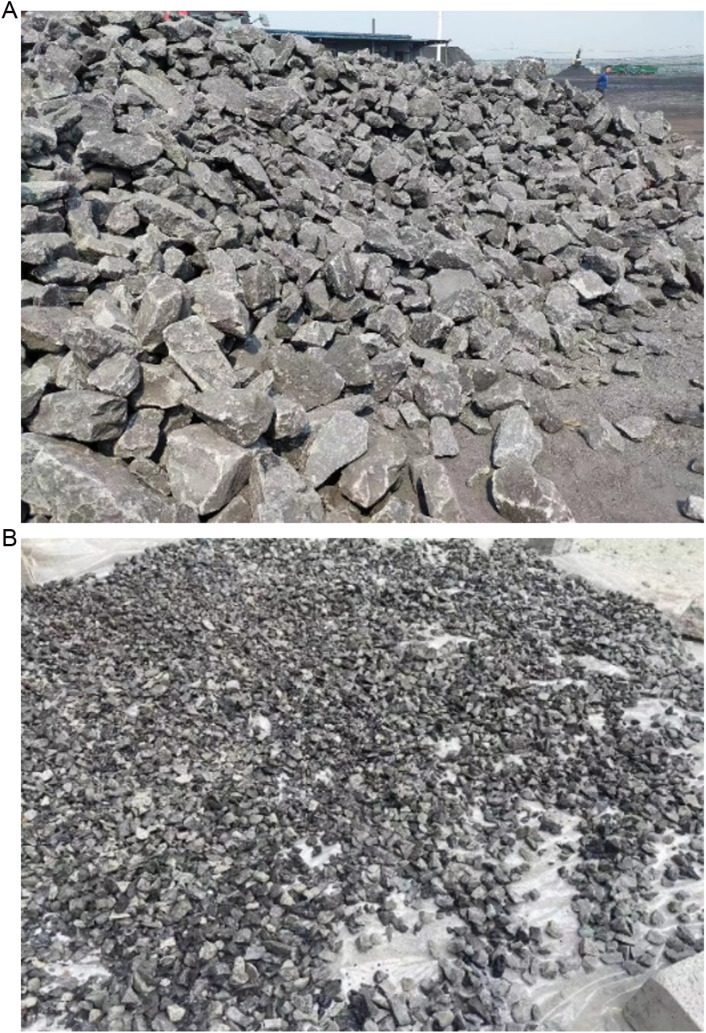
Raw coal gangue and coal gangue aggregates. (a) Raw coal gangue. (b) Coal gangue aggregates.

The coal gangue used in the test was collected from Jiudaoling Mine, Jinzhou, Liaoning Province. Since the coal gangue raw stones was too large, it was firstly broken by a jaw crusher. Stones with a diameter greater than 20mm were then manually knocked with a small hammer and finally screened using a sieve. The coal gangue raw stones and the coal gangue aggregate obtained after screening are shown in Fig 2. Additionally, the crushed stone and river sand used in the samples were provided by local suppliers, and Bohai brand ordinary Portland cement and laboratory clean tap water were used for sample preparation. GFRP was used as reinforcement material, the thickness was 0.114mm, the ultimate tensile strength was 526.85MPa, and the elastic modulus was 28.89GPa.

### 2.2 Specimen design

**Fig 3:**
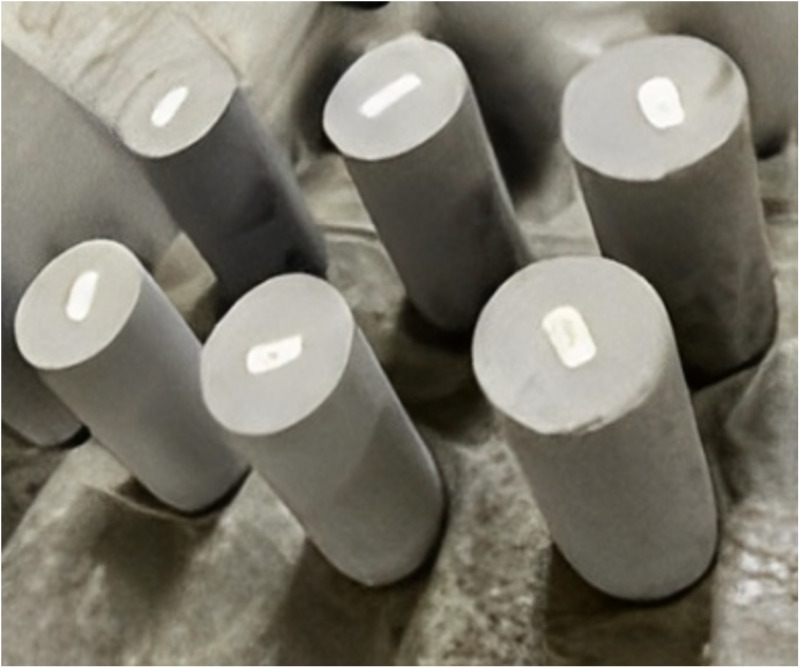
Coal gangue concrete specimens.

In this study, a total of 6 coal gangue concrete specimens were prepared (Fig 3), and the effects of GFRP confinement and coal gangue replacement rates (0%, 50% and 100%) on the axial compression properties of the specimens were evaluated. The specimens are all 150mm in diameter and 300mm in height. Two sets of parallel blocks were used under the same confinement and replacement rate. The specific naming rules of the sample are as follows: it first begins with the letter ‘F’ to represent the FRP confined specimen, the subscript ‘S’ and ‘M’ denote the test and simulation, respectively. The number ‘2’ represents the number of FRP layers, the capital letter ‘C’ in the third part represents CGC, while the numbers ‘0’, ‘50’ and ‘100’ represent the coal gangue replacement rate. Finally, roman numbers (I and II) are used at the end of the name to distinguish two identical parallel specimens. For example, ‘F-2-C50-Ⅱ’ represents the second concrete specimen confined by two layers of FRP with 50% coal gangue replacement rate.

### 2.3 Sample preparation

**Fig 4:**
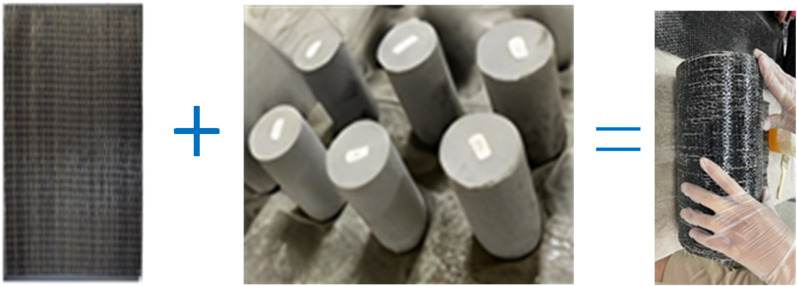
GFRP confined concrete specimens.

The specific production process of the sample is as follows: (1) The GFRP sheets were cut to match the specimen height (*H* = 300mm). The cloth length L is calculated as (*n*π+1) *D*, where *D* is the sample diameter (*D* = 150mm) and *n* is the number of winding layers of the GFRP sheet. Therefore, the sheet length used in this study is 1092mm. (2) Before wrapping the specimen with FRP sheets, the concrete surface is wiped to remove contaminants. (3) Apply glue to the pre-cut GFRP sheet. (4) Lay the GFRP sheet flat on the table, and wrap it around the side of the concrete specimen in a clockwise direction until the GFRP is perfectly fitted with the concrete without air bubbles. (5) The wrapped sample will be cured under laboratory conditions for 7 days. The prepared GFRP confined concrete cylinder is shown in Fig 4, and the detailed specimen information is presented in Table 1.

**Table 1:** Test results of GFRP confined concrete specimens.

Samples	$f_{c0}/MPa$	$f_{cu}/MPa$	$\varepsilon_{c0}$	$\varepsilon_{cu}$	*E*/GPa	$f_{cu}/f_{c0}$	$\varepsilon_{cu}/\varepsilon_{c0}$
F-0-C0	44.98		0.002		22.490		
F_S_-2-C0-Ⅰ		73.35		0.018	4.0750	1.63	9
F_S_-2-C0-Ⅱ		69.70		0.015	4.6467	1.55	7.5
F-0-C50	37.17		0.002		1.8585		
F_S_-2-C50-Ⅰ		55.13		0.011	5.0118	1.48	5.5
F_S_-2-C50-Ⅱ		50.15		0.008	6.2688	1.35	4
F-0-C100	28.73		0.002		14.365		
F_S_-2-C100-Ⅰ		42.67		0.010	4.2670	1.49	5
F_S_-2-C100-Ⅱ		43.61		0.009	4.8456	1.52	4.5

### 2.4 Test setup and instrumentation

To reveal the influence mechanism of FRP type and coal gangue replacement rate on the macro-mechanical behavior of concrete, uniaxial compression tests were conducted on FRP confined coal gangue concrete specimens in this study. The microcomputer-controlled electro-hydraulic servo rock triaxial testing machine (SAM-2000) produced by Changchun Kexin testing machine Co., Ltd was used in this test. Its loading system consists of four parts, including the test host, the oil source, the measurement and control system, and the test apparatus. The maximum axial pressure that can be exerted is 2000 kN. The DH3816 static strain tester (100Hz) from Donghua test was used to collect strain, stress, and displacement data during the experiment. Before the test, a small amount of lubricant was applied to both ends of the specimen to reduce the effect of end friction. Secondly, the sample was placed horizontally on the center of the pressure platform. Finally, the compression test was carried out. Based on the reference [36–39], the test was conducted by displacement control method with a loading rate of 0.6 mm/min, applying continuous loading until the specimen was damaged.

### 2.5 Test results

Table 1 presents the main test results of GFRP confined coal gangue concrete specimens, including peak stress (*f*_cu_), peak strain (*ε*_cu_), elastic modulus (*E*), stress enhancement ratio (*f*_cu_*/f*_c0_), and strain enhancement ratio (*ε*_cu_*/ε*_c0_). As can be seen from *f*_cu_*/f*_c0_ and *ε*_cu_*/ε*_c0_ in Table 1, the peak stresses and peak strains of GFRP confined concrete specimens are significantly increased compared to unconfined specimens at the same coal gangue replacement rate. This indicates that GFRP confinement can enhance the compressive strength of concrete and improve its deformation capacity. It is evident that both the peak stress and peak strain of the unconfined and confined specimens decrease as the coal gangue replacement rate increases. This suggests that the increase in coal gangue replacement rate reduces the confinement effect of GFRP, thereby weakening the compressive strength and ductility of the concrete.

## 3 Model introduction

### 3.1 Linear contact model and parallel bonding model

**Fig 5:**
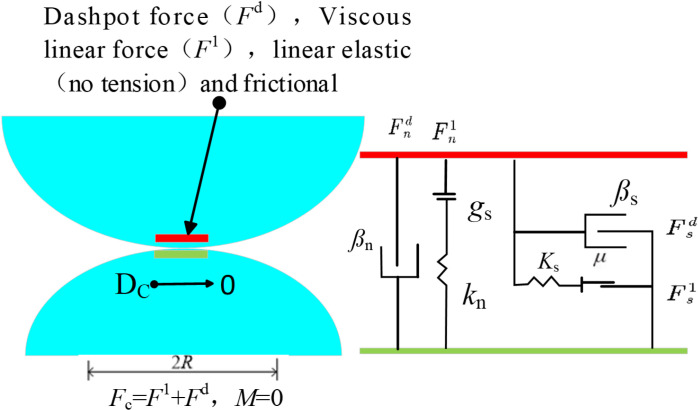
Linear contact model.

**Fig 6:**
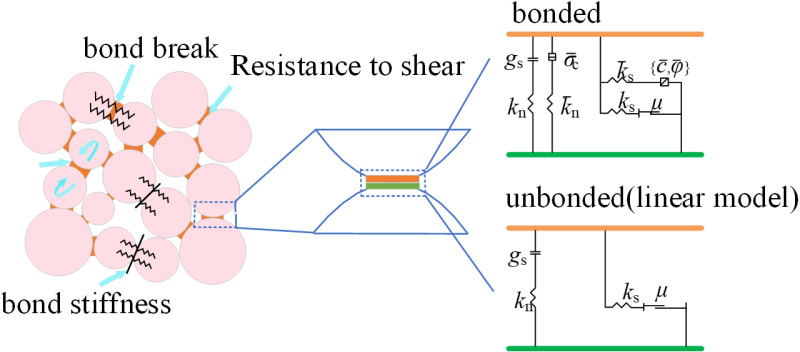
Parallel bonding model.


In PFC simulation, the linear contact model (Fig 5) and the parallel bonding model (Fig 6) were used to characterize the mechanical behavior between particles and between particles and walls. The linear contact model defines the elastic relationship between the contact force and the relative displacement, with both particle-particle and particle-wall contacts can be regarded as the interaction between springs. The linear model consists of a linear part and a damped part that act in parallel with each other. The linear part provides linear elastic and frictional behavior, and the damped part provides viscous behavior. The linear force is generated by a linear spring (*k*_n_, *k*_s_), which cannot maintain the tension, and the coulomb criterion is applied to the shear force by the friction coefficient *μ* to satisfy the slip condition. The linear stiffness is divided into tangential and normal stiffness, with the calculation formulas are as follows:

$$k_{n}=AE^{*}/L,k_{s}=k_{n}/k^{*}$$ (1)

$$\begin{array}{c} A=\left\{ \;\;\;\begin{array}{c} 2rt,2D(t=1)\\ \pi r^{2},3D \end{array}\right. \end{array}$$ (2)

$$\begin{array}{c} r=\left\{ \;\;\;\begin{array}{c} \min(\;\;R^{\left(1\right)},R^{\left(2\right)}),ball-ball\\ R^{\left(1\right)},ball-facet \end{array}\right. \end{array}$$ (3)

$$\begin{array}{c} L=\left\{ \;\;\;\begin{array}{c} R^{\left(1\right)}+R^{\left(2\right)},ball-ball\\ R^{\left(1\right)},ball-facet \end{array}\right. \end{array}$$ (4)

Potyondy and Cundall [40] proposed the parallel bonding model (PBM), which assumes that the bonding between particles is adhesive. In this model, the contact between two particles consists of a linear part and a bonded part. The linear part cannot resist relative rotation, and the slip is adjusted by applying coulomb limit to the shear force. When the particles rotate against each other to generate torque, the bonded part can resist the torque while transmitting force and moment, and behaves in a linear elastic manner until the force exceeds the strength limit, at which point the bonded model breaks down. The PBM is shown in Fig 6. The meanings of parameters in the figure are similar to those in the linear contact model, with the parameters of the bonded part are represented by the upper line. In the figure, *σ*_c_ is the tensile strength, *c* is the cohesion force, and *φ* is the internal friction angle. The stiffness calculation of the linear part in the parallel bonding model is the same as that of the linear contact model. Additionally, the stiffness of the bonded part is divided into tangential and normal stiffness, with the following calculation formulas:


$$\begin{array}{c} {\overset{―}{k}}_{n}={\overset{―}{E}}^{*}/L,{\overset{―}{\;k}}_{s}={\overset{―}{k}}_{n}/{\overset{―}{k}}^{*} \end{array}$$ (5)

$$\begin{array}{c} L=\left\{ \;\;\;\;\begin{array}{c} R^{\left(1\right)}+R^{\left(2\right)},ball-ball\\ R^{\left(1\right)},ball-facet \end{array}\right. \end{array}$$ (6)

### 3.2 PFC3D-FLAC3D coupling principle

**Fig 7:**
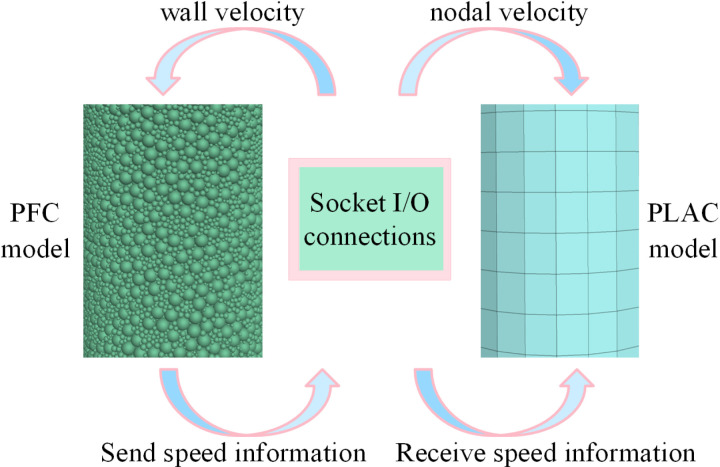
Principle of FLAC-PFC coupling calculation.

Shu et al. [41] employed point cloud analysis combined with machine learning techniques to achieve automated identification and measurement of reinforcement cages and corrugated pipes. Similarly, in this study, the PFC3D-FLAC3D numerical simulation is used to analyze the evolution of mesoscopic structures through particle tracking technology. Specifically, the particle flow code PFC3D is primarily used to simulate the micromechanical behavior of discontinuous media composed of rigid particles. Meanwhile, the finite difference program FLAC3D is widely used in slope stability evaluation, support design and evaluation, tunnel engineering and many other fields. FLAC3D can be run as a plug-in in PFC3D6.0, allowing the two programs to be coupled with force and moment data, as well as element spatial information. To better reflect the characteristics of FRP confined coal gangue concrete columns, the coupling analysis method of structural unit and PFC particles is adopted in this paper. Fig 7 shows the PFC-FLAC coupling principle. The velocity obtained from FLAC is transmitted to the PFC model through the socket connection, which then updates the response of PFC model. Subsequently, the force generated by the PFC model at the coupled boundary is fed back to the FLAC model, serving as boundary conditions for updating the mechanical response of the FLAC model. The above iterative process is repeated until both the FLAC and PFC models satisfy the equilibrium conditions and the coupling analysis is terminated.

## 4 Numerical simulation

### 4.1 Numerical modeling

**Fig 8:**
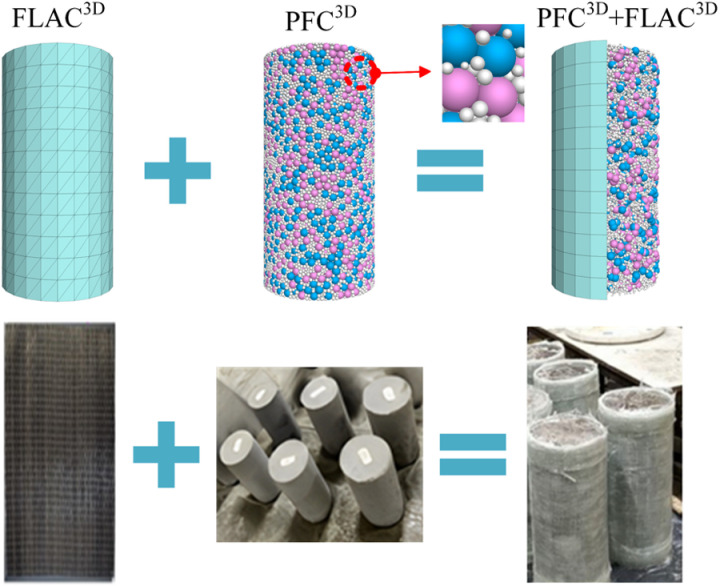
FRP sheets wrapped specimen.

The GFRP confined coal gangue concrete cylinder model has the same size as the experimental coal gangue concrete cylinder, with a diameter of 150mm and a height of 300mm. The model contains three different colors and sizes of particles, with pink, blue, and white to represent coal gangue, coarse aggregate, and fine aggregate, respectively. The thickness of the FRP sheet is 0.114mm and the elastic modulus is 11.6GPa. For reference to the coal cylinder PFC3D model in literature [42–43], the coal gangue concrete specimen consists of 78113 particles with radius uniformly distributed from 0.8mm to 5mm, as shown in Fig 8.

**Fig 9:**
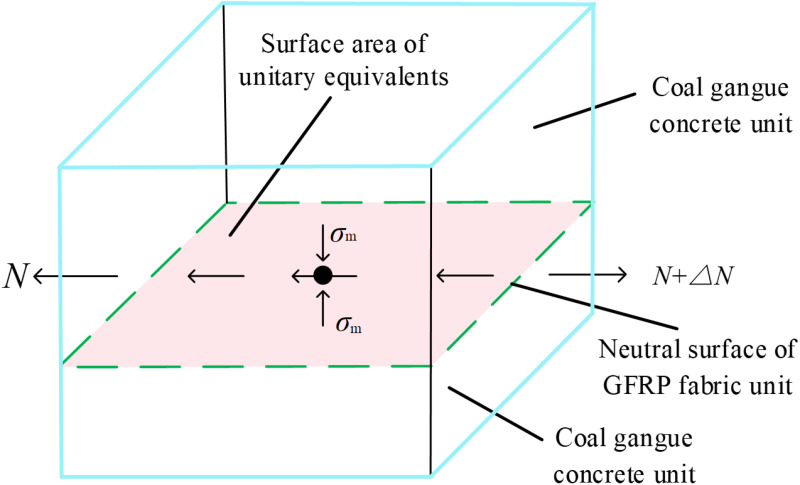
Mechanical model of geogrid element.

In the numerical simulation test, a light-colored geogrid with a thickness of 0.114mm was used to simulate the FRP sheet. In uniaxial compression, the FRP sheet is an anisotropic linear elastic material, which can only bear transverse tensile forces. The mechanical model is shown in Fig 9. The lateral restraint force *σ*_m_ and the total shear stress *τ* together constitute the stress on the geogrid structure, while the membrane stress N inside the geogrid element plays a balancing role. *σ*_m_ is the normal stress applied to the surface of the unit, and the stress of the geogrid can be analyzed by the color change and deformation of the surface. During the simulated loading test, the roof was fixed and a loading rate of 0.6mm/min was applied to the loading plate. The average stress and displacement at the end are recorded to obtain the overall axial stress-strain curve of the FRP confined coal gangue concrete cylinder.

### 4.2 Parameter calibration

**Fig 10:**
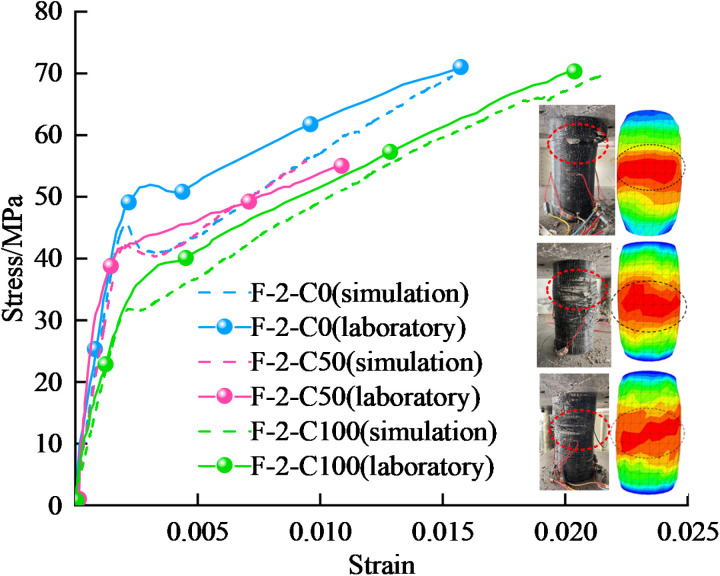
Comparison of experimental and simulation results of GFRP confined specimens.

In the process of numerical simulation, the calibration of mesoscopic parameters is the basic premise to ensure the accuracy and reliability of simulation results. To obtain reliable micro-parameters, uniaxial compression tests were conducted on GFRP confined coal gangue concrete specimens in the laboratory. To ensure the reliability of the calibrated parameters, the dimensions of the coal gangue concrete specimens, the coal gangue replacement rate, the loading rate, and the number of particles used in the simulation are kept consistent with those in the laboratory tests. In this paper, the ‘trial and error method’ was adopted to continuously adjust detailed mesoscale simulation parameters until the failure mode and stress-strain curve of the numerical simulation sample closely matched the results of the laboratory tests. The comparison of the failure mode and the stress-strain curve are shown in Fig 10.

As shown in Fig 10, the stress-strain curve and the failure mode of the specimens exhibit a high degree of agreement. The errors between the test and the simulation are presented in Table 2, with the relative errors in peak stress and peak strain both within 5%. This indicates that the parameters obtained from the numerical simulation of GFRP confined specimens under uniaxial compression are reliable. The detailed mesoscopic parameters of the PFC3D-FLAC3D coupling model are shown in Table 3. ‘Coal gangue-coal gangue’ refers to the contact between the coal gangue particles, ‘coarse aggregate-coarse aggregate’ represents the contact between the coarse aggregate particles, ‘fine aggregate-fine aggregate’ denotes the contact between the fine aggregate particles, and the ‘Ball-facet’ refers to the contact between the particle and the wall.

**Table 2:** Relative error between test and simulation results for GFRP confined specimens.

Samples	Peak stress/MPa		Peak strain
F-2-C0	Test	71.525	0.0165
	Simulation	71.06	0.0160
	Relative error	0.65%	0%
F-2-C50	Test	52.64	0.0095
	Simulation	56.07	0.0019
	Relative error	6.12	5%
F-2-C100	Test	43.14	0.0095
	Simulation	43.26	0.0095
	Relative error	0.3	0%

**Table 3:** Mesoscopic parameters of PFC3D-FLAC3D coupling model.

Parameter type	Name	Value	Unit	Parameter symbol
Coal gangue-coal gangue	Stiffness ratio	1.0	/	*k*
	Friction coefficient	0.513	/	*μ*
Coarse aggregate-coarse aggregate	Contact modulus	24	GPa	*E*
	Stiffness ratio	1.0	/	*k*
	Friction coefficient	0.57	/	*μ*
Fine aggregate-fine aggregate	Contact modulus	24	GPa	*E*
	Stiffness ratio	1.0	/	*k*
	Normal bond strength	53	MPa	**σ*c*
	Tangential bond strength	53	MPa	*c*
	Friction angle	25		*Φ*
	Friction coefficient	0.57	/	*μ*
Ball-facet	Contact modulus	24	GPa	*E*
	Stiffness ratio	1.0	/	*k*
	Friction coefficient	10	/	*μ*
	Particle density	2700	kg/m^3^	*ρ*
Geotextile	Elastic modulus	100	GPa	*E*
	Poisson’s ratio	0.33	/	*μ*

### 4.3 Numerical simulation scheme

Based on the uniaxial compression test of GFRP confined coal gangue concrete, the loading rate for the numerical simulation of the uniaxial compression test is set to 0.6mm/min. According to the principle of orthogonal test, the orthogonal scheme was designed for the sensitivity factors of FRP sheet type and coal gangue substitution rate. The study investigated the influence of these sensitivity factors on the axial compression performance of coal gangue concrete columns. The details of the microscopic simulation scheme are shown in Table 4.

**Table 4:** Numerical simulation scheme.

FRP type	FRP layers	Coal gangue replacement rate
GFRP	2	0%
		50%
		100%
CFRP	2	0%
		50%
		100%
BFRP	2	0%
		50%
		100%

## 5 Results and discussion

### 5.1 Stress-strain curves

**Fig 11:**
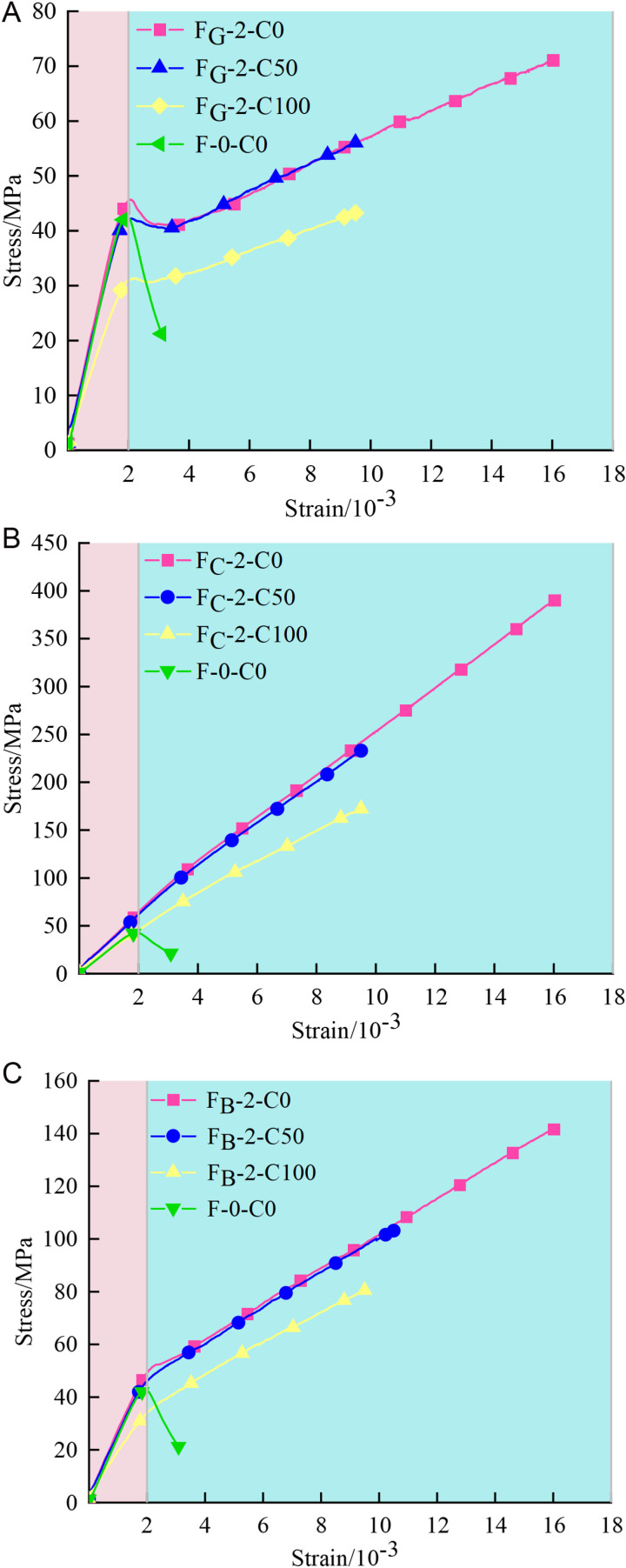
Stress-strain curves of FRP confined specimens. (a) GFRP confined specimens. (b) CFRP confined specimens. (c) BFRP confined specimen.

The axial stress-strain curves of FRP confined coal gangue concrete cylinders under different conditions from the meso simulation are shown in Fig 11. For better comparative analysis, the unconstrained concrete columns are also drawn in the Figure. It is worth noting that in the numerical simulation part of this paper, the peak strain of GFRP confined coal gangue concrete columns was used as the stopping condition, while the peak strain of CFRP and BFRP confined coal gangue concrete columns are predicted purely through simulation. To facilitate the analysis, the peak strain of GFRP confined coal gangue concrete columns is still used as the stopping condition.

In general, the axial stress-strain curves of the GFRP/CFRP/BFRP confined coal gangue concrete cylindrical specimens are similar, all exhibiting a bilinear variation with a smooth transition zone at the peak stress of the unconfined specimen. This behavior is consistent with the findings of Zhao [44] and Wang [28] et al. In the initial ascending phase, as shown in the pink section of the figure, the external FRP is not yet activated and thus does not constrain the internal specimens. As a result, the behavior of the FRP confined specimen closely resembles that of the unconfined concrete specimen, with the same slope in the stress-strain curves. In the transition phase, the peak stress of the concrete wrapped with two layers of FRP is significantly higher compared to the unconfined sample. This is due to the circumferential confinement effect of the FRP, which restricts the lateral deformation of the concrete and enhances the interaction forces between the particles, thereby improving its overall compressive strength. As the number of confinement layers increases, the circumferential confinement force also enhanced, effectively suppressing the initiation and propagation of internal cracks in the concrete under compression, thus enhancing its load-bearing capacity. Moreover, in the second phase (the blue section in the figure), the stress-strain curves of the unconfined specimen drops rapidly, exhibiting apparent brittleness. In contrast, the stress of specimen wrapped with two layers of FRP continues to rise, allowing it to sustain a certain load even under large deformation, demonstrating excellent ductility. This is because the FRP continues to provide confinement after the concrete cracks, delaying crack propagation and improving the deformation capacity of the concrete. These results indicate that FRP materials play a crucial role in enhancing the compressive strength and ductility of concrete structures, especially in the design of high-strength and high-toughness structures, where they can offer more durable and stable support to the concrete.

Through careful observation, it can be found that the stress-strain curves of GFRP confined specimens generally exhibit distinct inflection points, indicating pronounced strain hardening behavior. In the initial stage, stress increases rapidly with strain, resulting in a steep slope. Subsequently, the slope of the curve gradually decreases, the rate of stress growth slows down, and the specimen enters the plastic stage, exhibiting noticeable ductility. For CFRP confined specimens, the stress-strain curves maintain a stable growth trend with minimal variation in slope, reflecting a smooth elastic-plastic transition characteristics. These specimens demonstrate high strength and excellent ductility, with the highest ultimate stress values. The stress-strain curves of BFRP confined specimens also have inflection points, though the inflection point phenomenon is not as obvious as that of GFRP confined specimens. In the initial phase, stress rises sharply, characterized by a steep slope. Subsequently, the slope gradually decreases, and the stress growth rate slows down, yet it continues to rise until reaching a relatively high stress level, showing a certain degree of ductility.

**Fig 12:**
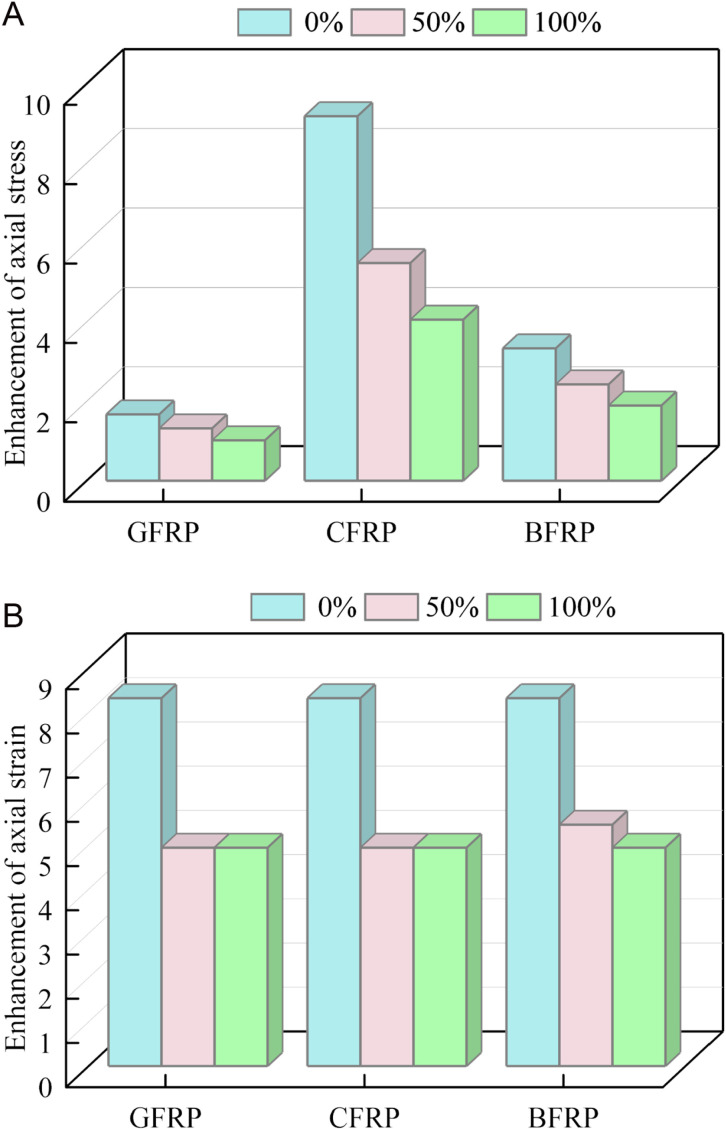
Enhancement of stress and strain for FRP confined specimens. (a) Stress. (b) Strain.

To further evaluate the confinement effect of FRP on coal gangue concrete columns, Fig 12 sequentially presents the stress-strain improvement of FRP confined coal gangue concrete specimens. The vertical axis of Fig 12(a) represents the ratio of the peak stress of FRP confined specimens to that of unconfined specimens. Both the strength and ductility of the specimen were significantly improved with FRP confinement. It can be clearly seen from Fig 12(a) that when the coal gangue replacement rate is 0%, the stress of the GFRP, CFRP, and BFRP confined specimens increased by 1.67, 9.19, and 3.34 times, respectively, compared to the unconfined specimens. When the coal gangue replacement rate is 50%, the stress of the GFRP, CFRP, and BFRP confined specimens increased by 1.32, 5.49, and 2.43 times, respectively, compared to the unconfined specimens. As the coal gangue replacement rate continues to increase, the confinement effect of FRP on the specimens decreases. When the coal gangue replacement rate reaches 100%, the stress of the GFRP, CFRP, and BFRP confined specimens increased by only 1.02, 4.06, and 1.89 times, respectively, compared to the unconfined specimens. This shows a significant difference compared to the 0% and 50% coal gangue replacement cases, which is primarily due to the lower strength of coal gangue. Comparing the stress improvement ratios under the three types of confinement, the stress enhancement varies significantly, with the confinement effect in the order of CFRP > BFRP > GFRP.

The vertical axis of Fig 12(b) is the ratio of the axial strain corresponding to the peak stress of the FRP confined specimen to that of the unconfined specimen. It is obvious that the axial strain enhancement ratio of the three FRP confined specimens is the largest when the coal gangue replacement rate is 0%. In contrast, the strain enhancement ratios of the specimens with 50% and 100% replacement rate are greatly reduced, with a relatively small difference between the two. Furthermore, the improvement in axial stress induced by different FRP types on specimens is basically the same. This is due to the above mentioned, CFRP and BFRP confined coal gangue concrete columns are pure simulation predictions. To facilitate the analysis, the peak strain for GFRP confined coal gangue concrete column was used as the stopping condition.

### 5.2 Compressive strength

**Fig 13:**
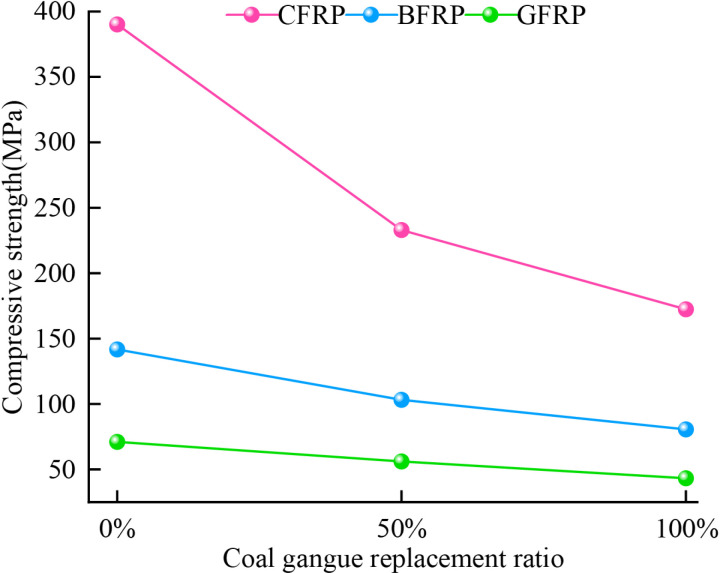
Compressive strength of FRP confined CGC.

Fig 13 shows the compressive strength of GFRP, CFRP, and BFRP confined concrete specimens under different coal gangue aggregate replacement rates. Overall, whether it is GFRP, CFRP, or BFRP confined concrete specimens, the compressive strength of the concrete is relatively low at 50% and 100% coal gangue replacement rates, which is consistent with the research results of Wang [5] et al. This indicates that, under the same number of FRP confinement layers, the compressive strength of the specimens decreases as the coal gangue replacement rate increases, which is primarily due to the lower strength of coal gangue compared to traditional coarse aggregates.

For CFRP confined specimens, the compressive strength under 0%, 50% and 100% coal gangue replacement ratio are 390.14MPa, 233.01MPa and 172.42MPa, respectively. Compared to the FC-2-C0 specimens with 0% replacement rate, the strength of concrete under 50% and 100% coal gangue replacement rate decreased by 40% and 55% respectively. For BFRP confined specimens, the compressive strength under 0%, 50% and 100% coal gangue replacement rate are 141.69Mpa, 103.17Mpa and 80.55MPa, respectively. Compared with the FB-2-C0 specimens, the strength of concrete under 50% and 100% coal gangue replacement ratio decreased by 27% and 43% respectively. For GFRP confined specimens, the compressive strength under 0%, 50% and 100% coal gangue replacement rate are 71.06MPa, 56.07MPa and 43.26MPa, respectively. Compared with the FG-2-C0 specimens, the strength of concrete under 50% and 100% coal gangue replacement rate decreased by 21% and 39% respectively.

Based on the above data, it is evident that under different FRP confinements, the compressive strength of concrete decreases significantly as the coal gangue replacement rate increases. Firstly, the primary factor behind this trend is the lower strength of coal gangue compared to coarse aggregates. In the concrete structure, aggregates are crucial for load transfer. When coal gangue replaces coarse aggregates, due to its low strength, the ability of stress transfer is weakened. With the increase in the replacement rate of coal gangue, the proportion of weak regions in the concrete rises, making the structure more susceptible to cracking and damage, thereby reducing the compressive strength. Additionally, as the coal gangue replacement rate increases, the internal structure of the concrete undergoes significant changes. Differences in the particle shape and gradation of coal gangue lead to increased porosity and a looser structure, which in turn reduces the density and compressive strength of the concrete. Meanwhile, the poor bonding performance between coal gangue and the cement paste makes the concrete interface more prone to debond, further damaging the internal structure and causing a significant decrease in compressive strength. Although FRP confinement can enhance compressive strength by restricting lateral deformation and strengthening the interaction between internal particles, this effect diminishes as the level of confinement decreases. As a result, cracks and damage are more likely to occur within the concrete, further reducing its load-bearing capacity. Consequently, the concrete becomes inability to effectively withstand external loads, leading to a significant decline in the overall performance.

With its high elastic modulus and excellent tensile properties, CFRP provides strong confinement for concrete and effectively maintains the stability of concrete structures. It significantly restricts the lateral deformation of concrete under compression, thereby greatly enhancing its compressive strength. Furthermore, the interfacial bonding performance between CFRP and concrete is excellent, which can efficiently transfer external loads to concrete and further improve its overall stiffness and compressive strength. The material properties of BFRP enable it to provide some confinement to concrete. However, its elastic modulus and tensile strength are lower and the bonding performance of the interface with concrete is weaker compared with CFRP. Therefore, its effect in enhancing the strength of concrete is not as significant as that of CFRP. Due to its material properties, GFRP provides lower rigidity and load-bearing capacity in confining concrete. Moreover, its bonding performance with coal gangue concrete is relatively weak, resulting in reduced load transfer efficiency. Consequently, the enhancement effect of GFRP in improving the compressive strength of concrete is limited.

In conclusion, the compressive strength of CFRP confined specimens is the highest under any replacement rate, which is significantly exceeding that of the BFRP and GFRP confined specimens. The order of confinement strength is CFRP > BFRP > GFRP. This is differs from the findings of Abed [45], where the performance differences between BFRP and GFRP are mainly attributed to variations in their interface bonding with concrete, the properties of coal gangue concrete, the different loading rates and the influence of temperature [46] on the compressive strength of concrete. BFRP may improve the compressive strength of concrete through a stronger confinement effect.

### 5.3 Elastic modulus

**Fig 14:**
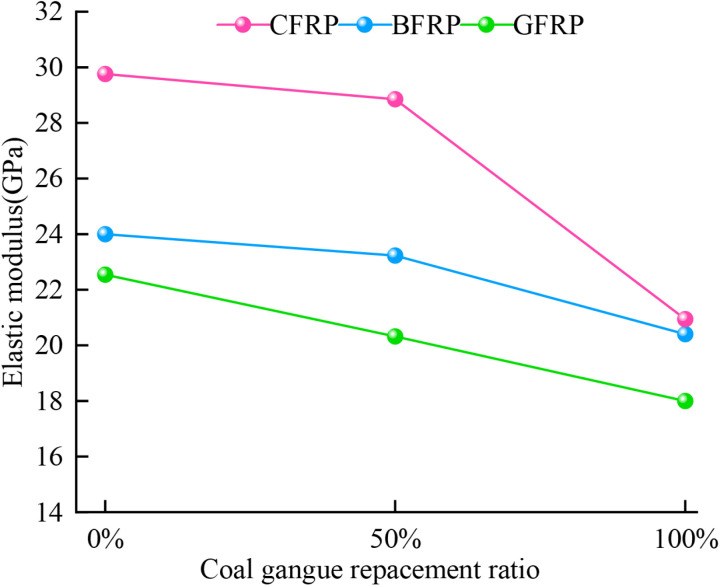
Elastic modulus of FRP confined CGC.

The variation curve of the elastic modulus of FRP confined specimens with the coal gangue substitution rate is shown in Fig 14. It is not difficult to find that the elastic modulus of FRP confined coal gangue concrete decreases as the coal gangue replacement rate increases, which is consistent with the research results of Zhou [47] et al. In general, the curve of the elastic modulus versus the coal gangue replacement rate can be divided into two phases. In the phase of low coal gangue replacement rate (0% - 50%), the elastic modulus decreases gradually as the replacement rate increases. Even though the addition of coal gangue has a certain impact on the internal structure and properties of concrete, it does not significantly affect the stability of the internal structure and elastic properties of concrete under the dominant role of ordinary coarse aggregate. Therefore, the weakening effect on the overall elastic performance is relatively limited, and the decline in elastic modulus remains gradual. In the phase of high coal gangue replacement rate (50% - 100%), the decrease in elastic modulus increases significantly. This is primarily due to the significant increase in the content of coal gangue, which leads to significant changes in the internal structure and performance of the concrete. Firstly, the irregular particle shape of coal gangue, combined with its different gradation compared to ordinary coarse aggregates, leads to significant change in the accumulation of particles inside the concrete. Irregular geometric surfaces and sharp polyhedral aggregates are more prone to accumulate damage compared to conventional aggregates [48], which in turn affects the overall mechanical performance of the concrete. As the coal gangue replacement rate increases, the internal pores increase and the structure becomes more porous. During the process of loading, these additional internal pores will deform and consume more energy, which drastically reduces the ability of concrete to resist elastic deformation, ultimately leading to a decrease in the elastic modulus. Secondly, the relatively poor bond between coal gangue and the cement paste becomes more evident at higher replacement rates. This hinders stress transfer within the concrete, further weakening its elastic properties and resulting in a further reduction in elastic modulus.

For CFRP confined specimens, the elastic modulus under 0%, 50% and 100% coal gangue replacement rates are 29.76 GPa, 28.85 GPa, and 20.94 GPa, respectively. Compared to the FC-2-C0 specimen with 0% replacement rate, the elastic modulus of concrete with 50% and 100% replacement rates decreased by 3.1% and 30%, respectively. For BFRP confined specimens, the elastic modulus under 0%, 50% and 100% coal gangue replacement rate are 25.64GPa, 23.11GPa and 15.22GPa, respectively. Compared to the FB-2-C0 specimen, the elastic modulus of concrete with 50% and 100% replacement rates decreased by 3.2% and 15%, respectively. For GFRP confined specimens, the elastic modulus of 0%, 50% and 100% coal gangue replacement rate are 26.94GPa, 23.78GPa and 16.75GPa, respectively. Compared to the FG-2-C0 specimen, the elastic modulus of concrete under 50% and 100% replacement rates decreased by 9.8% and 20% respectively. Although FRP confinement can improve the deformation resistance of concrete, the enhancement effect of FRP is limited at high coal gangue replacement rate. This is due to the significant decrease of concrete strength and stiffness, which leads to a significant decrease in elastic modulus and bearing capacity. As the confinement effect weakens, the restriction on lateral deformation of the concrete decreases, making it more prone to cracks and damage under loading. This ultimately leads to a reduction in the overall elastic modulus. In addition, the CFRP confined specimens had the highest elastic modulus at any replacement rate, which was significantly higher than that of the GFRP and BFRP confined specimens. The confinement strength is ranked as follows: CFRP > BFRP > GFRP.

### 5.4 Contact number and contact force

**Fig 15:**
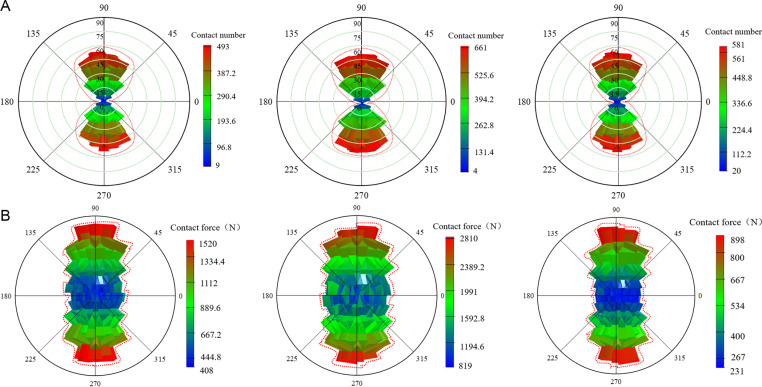
The number of strong contact and strong contact force of GFRP confined concrete specimens under three coal gangue replacement rates (0%, 50%, 100%). **(a)** Strong contact number. **(b)** Strong contact force.

**Fig 16:**
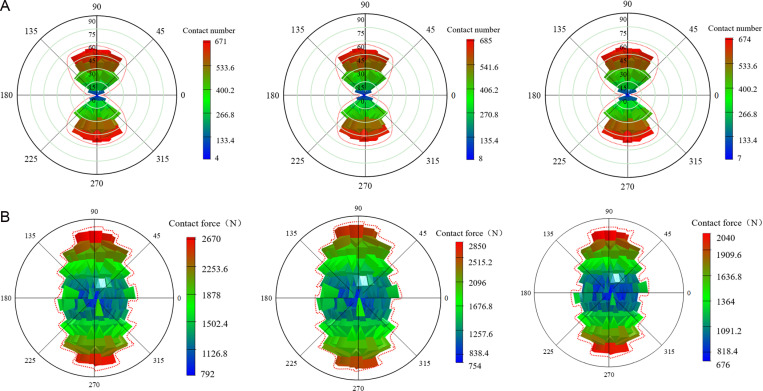
The number of strong contact and strong contact force of CFRP confined concrete specimens under three coal gangue replacement rates (0%, 50%, 100%). **(a)** Strong contact number. **(b)** Strong contact force.

**Fig 17:**
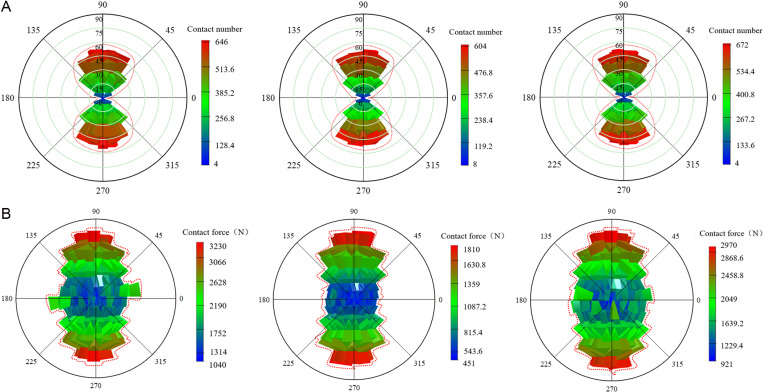
The number of strong contact and strong contact force of BFRP confined concrete specimens under three coal gangue replacement rates (0%, 50%, 100%). **(a)** Strong contact number. **(b)** Strong contact force.

To better understand and predict the macroscopic mechanical properties of materials, a statistical analysis was conducted on the number of strong contact and the corresponding contact force in coal gangue concrete specimens under different types of FRP confinement. This analysis provides a deeper investigation into the effects of coal gangue replacement rate and FRP type on the axial compressive properties of the concrete specimens. The principle involves dividing the surface of a standard sphere into 320 triangular grid regions of equal area. The column height directly reflects the dynamic change of strength, with the column height is proportional to the warmth of the color. In other words, the higher the height of the column, the warmer the color, which means the more concentrated the contact distribution and the greater the contact force. Figs 15–17 show the spatial distribution of strong contact number and strong contact force of different coal gangue concrete under GFRP, CFRP and BFRP confinement. For the convenience of description, the contact with a lower value is defined as the general contact force, and the force chain higher than the average value of all force chains is defined as the strong contact force.

**Fig 18:**
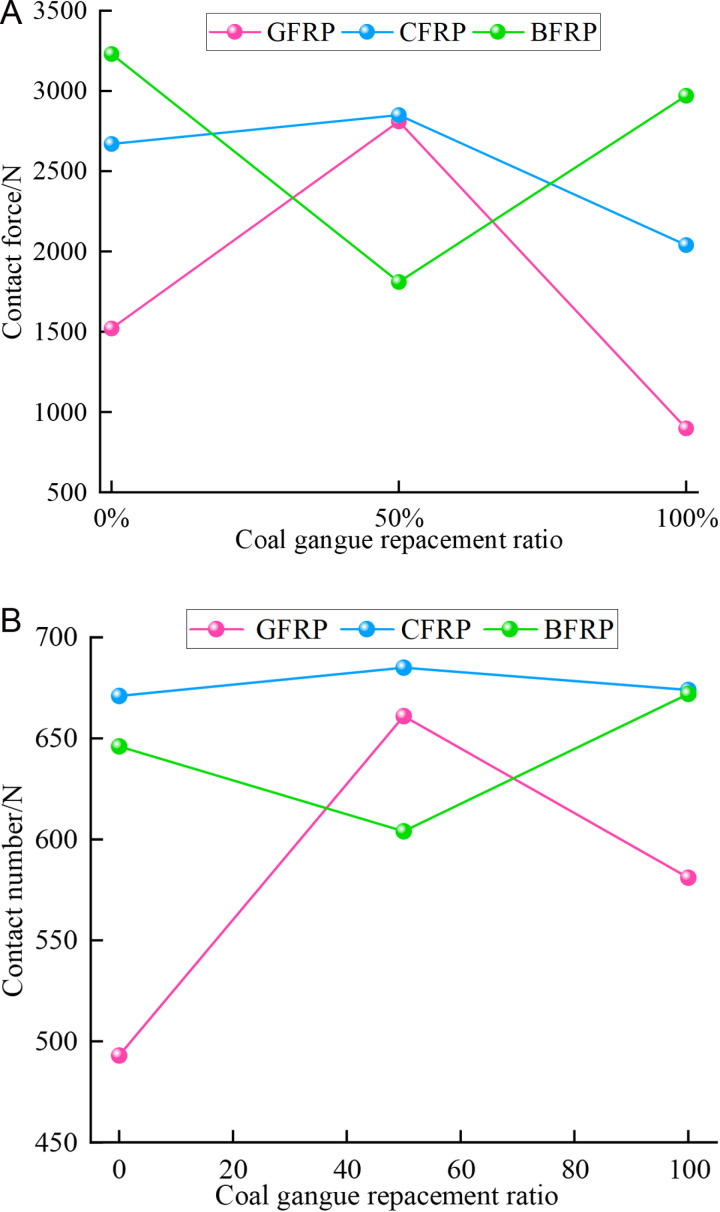
The maximum values of strong contact force and strong contact number for FRP confined CGC. **(a)** Strong contact force. **(b)** Strong contact number.

To visually observe the distribution of strong contact force and the number of strong contact in FRP confined concrete specimens under different coal gangue replacement rates, the maximum values of strong contact force and the number of strong contact for FRP confined concrete specimens are plotted, as shown in Fig 18. Based on Figs 15–18, it is evident that for GFRP confined specimens, the difference between the maximum and minimum contact number is small, indicating relatively low anisotropy and a more uniform distribution. It is clear that the distribution of strong contact number under each replacement rate exhibits a unique ‘peanut’ shape. Furthermore, it is apparent that the maximum values of both strong contact number and force are similar under each replacement rate, indicating the coal gangue replacement rate has a small effect on the number of strong contact. In terms of contact force, there is a significant variation across the three replacement rates, yet the distribution pattern of strong contact force is approximately ‘elliptical,’ with relatively high anisotropy. In contrast, the contact number for CFRP confined specimens increase dramatically, with the overall distribution also following a ‘peanut’ shape, characterized by larger values at the ends and smaller in the middle. Additionally, the contact force of the CFRP confined specimens is also significantly increased, exhibiting a generally ‘elliptical’ distribution but with lower anisotropy. For BFRP confined specimens, the contact number distribution of the specimens with different replacement rates is almost the same, maintaining the ‘peanut’ shape, featuring larger values at the ends and smaller in the middle. Compared to GFRP and CFRP confined specimens, the contact force for BFRP confined specimens further increased, displaying significant variations in contact force distribution, yet still maintaining an ‘elliptical’ pattern.

In summary, as the number of strong contact increases, the force chain connections within the concrete become tighter and richer, forming a more complex and efficient load transmission system. When subjected to external loads, this system ensures a more uniform stress distribution, significantly enhancing the overall bearing capacity of the specimen. In contrast, when the number of strong contact decreases, the connection strength of the internal structure is weakened, which can easily lead to the phenomenon of stress concentration, and ultimately reducing its overall bearing capacity. Additionally, the confining force between particles in the concrete is weakened, and the relative sliding and displacement between the particles are increased. This results in greater and unevenly distributed deformation, further reducing the deformation resistance of the specimen. This indicates that the internal microstructural force chain is closely related to the ductility of concrete. Therefore, in concrete engineering, by appropriately selecting aggregate gradation, controlling the coal gangue replacement ratio and other measures, the number of strong contact can be effectively increased, the force chain structure can be improved, and the ductility of the material can be enhance.

Fig 18 reveals that for GFRP and CFRP confined concrete specimens, both the strong contact force and the number of strong contact initially increase and then decrease as the coal gangue replacement rate increases. In contrast, for BFRP confined concrete specimens, both the strong contact force and the number of strong contacts decrease initially and then increase as the coal gangue replacement rate increases. This phenomenon can be attributed to two primary reasons. Firstly, as the coal gangue replacement rate increases from 0% to 50%, the high stiffness of CFRP and GFRP provides effective confinement, enhancing the overall load-bearing capacity of the specimen, leading to an increase in both contact force and the number of contacts. However, when the coal gangue replacement rate reaches 100%, the low strength and high porosity of coal gangue reduce the overall strength of the specimen, making it difficult for external confinement to counteract the degradation of material properties, with the contact force and number of contact decreased. Secondly, the stiffness of BFRP is lower, the internal structure of the specimen is not effectively confined when the replacement rate is between 0% and 50%, resulting in a reduction in contact force and contact number. As the replacement rate rises from 50% to 100%, the flexibility and ductility of BFRP gradually become apparent, which provided flexible constraints to the specimens during the large deformation stage. This helps control the deformation, leading to a recovery in both contact force and the number of contacts. This phenomenon suggests that different types of FRP materials have different effects on the ductility of concrete due to their stiffness and flexibility. In the design of concrete engineering, the appropriate FRP material should be selected based on the specific requirements of the project. CFRP or GFRP can be given priority in projects with high requirements for early strength and ductility. On the other hand, for structures that may experience significant deformation in later stage, BFRP can be used as an alternative material due to its flexible confinement during long-term deformation.

### 5.5 The crack evolution characteristics

**Fig 19:**
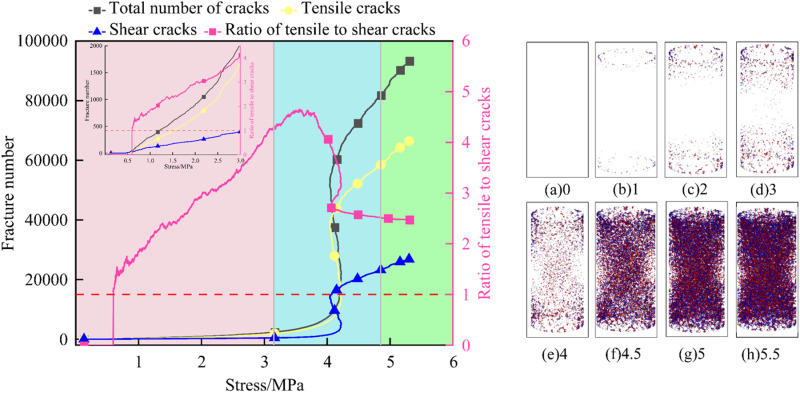
Crack propagation characteristics in the failure of GFRP confined coal gangue concrete.

**Fig 20:**
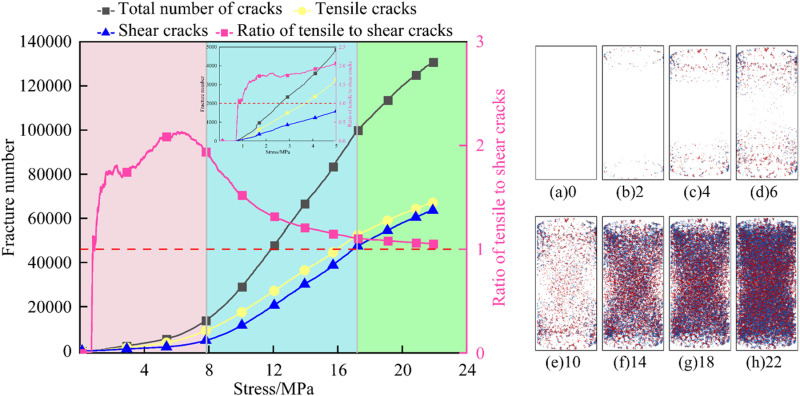
Crack propagation characteristics in the failure of CFRP confined coal gangue concrete.

**Fig 21:**
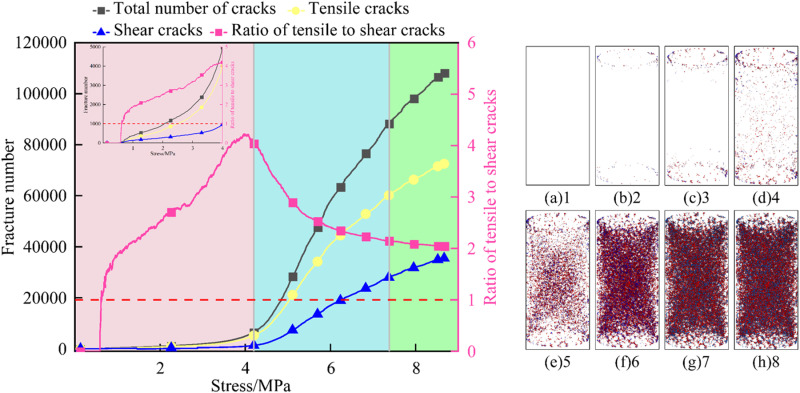
Crack propagation characteristics in the failure of BFRP confined coal gangue concrete.

To reveal the failure mechanism of FRP confined coal gangue concrete under uniaxial compression, it is crucial to study the crack development process during the loading process. This not only deepens the understanding of its mechanical behavior, but also effectively reduces maintenance costs and prevents catastrophic failures [49]. The concrete experienced the deformation and damage process of crack initiation, expansion and penetration during the loading process. Due to the large amount of data and the similar crack evolution law of concrete with different coal gangue replacement rates, this study takes the specimen with 50% coal gangue replacement rate as an example to analyze the failure mechanism of FRP confined coal gangue concrete from a microscopic perspective. Figs 19–21 sequentially show the variation curves of the total number of cracks, shear cracks, tensile cracks, and the ratio of tensile cracks to shear cracks over stress for GFRP, CFRP, and BFRP confined coal gangue concrete during the failure process, highlighting the crack evolution characteristics.

As illustrated in Fig 19, when the stress ranges from 0 to 1 MPa, the number of tensile and shear cracks within the GFRP confined coal gangue concrete structure gradually increases. The tensile cracks grow at a faster rate, but the shear cracks are more numerous. Consequently, the ratio of tensile to shear cracks increases linearly with the rising stress. As depicted in Fig 19(b), when the stress reaches 1 MPa, corresponding to the initial stage of the test, the application of stress leads to uneven local stress transfer due to the inherent porosity and heterogeneity of the concrete. As a result, micro-cracks are primarily concentrated at both ends of the concrete specimen. At this point, the number of tensile and shear cracks are 45 and 32, respectively.

When the stress ranges from 1 to 2 MPa, these cracks continue to expand as the stress increases gradually. However, the crack propagation rate is slower during the initial stage of loading, causing the micro-cracks to remain primarily concentrated in the local region at both ends of the structure, as shown in Fig 19(c). It can be seen from Fig 19(d) that when the stress increases to 3MPa, the cracks begin to propagate toward the center of the specimen. The rate of increase in tensile cracks is faster, which is further supported by the change in the ratio of tensile to shear crack with increasing stress.

When the stress reaches 4 MPa, the internal stress of the concrete structure approaches its tensile strength gradually, causing a sharp increase in the number of micro-cracks and the initial formation of a main crack, which generally exhibits an ‘X’ shape distribution. However, the number of tensile cracks is much higher than that of shear cracks, with the ratio of tensile to shear cracks around 4.5. As shown in Fig 19(e)-(h), with the increase in load, both tensile and shear cracks increase, merge, and gather rapidly. The interaction between these cracks promotes their coalescence into larger cracks, causing the main crack to widen quickly, and the ‘X’ shape distribution becomes more pronounced. Moreover, it is clear that tensile cracks continue to dominate throughout the process.

Fig 20 shows the relationship between the number of cracks and stress during the failure process of CFRP confined CGC specimens. As shown in the figure, when the stress is between 0 and 2 MPa, the development of micro-cracks within the concrete structure is relatively slow, with 108 tensile cracks and 103 shear cracks. When the stress reaches 4 MPa, the distribution of micro-cracks extends further toward the center of the specimen. The number of tensile cracks surpasses that of shear cracks, and the variation in the ratio of tensile to shear cracks further highlights this trend. It can be seen from Fig 20(d) that when the stress increases to 6MPa, the number of micro-cracks continues to rise, and their distribution expands further. The ratio of tensile to shear crack continues to increase. When the stress reaches 10 MPa, the main crack begins to form within the structure, with the number of tensile cracks significantly higher than shear cracks, and the ratio of tensile to shear is around 2.3. When the stress exceeds 10 MPa, both tensile and shear cracks propagate and coalesce rapidly, mainly distributed in the diagonal direction of the specimen, still exhibiting an ‘X’ shape distribution. During this process, the growth rate of tensile cracks is slower than that of shear cracks. The expansion and coalescence of these cracks reduce the local shear strength, hindering the effective transmission of shear stress, which results in a decrease in the ratio of tensile to shear cracks.

As shown in Fig 21, when the stress ranges from 0 to 2 MPa, the development of tensile and shear cracks within the BFRP confined coal gangue concrete structure is relatively slow, with tensile cracks being dominant. These cracks are concentrated at both ends of the specimen, and no micro-cracks appear in the center. When the stress reaches 3–4 MPa, the expansion rate of micro-cracks increases, and cracks begin to form in the center of the specimen. During this process, the ratio of tensile to shear cracks reaches its maximum value, close to 4.5. When the stress is 5MPa, a large number of micro-cracks appear in the center of the structure, and the overall distribution of cracks remains an ‘X’ shape. At this point, the influence of tensile cracks on structural failure becomes more pronounced, while the effect of shear cracks is comparatively weaker, leading to a decrease in the ratio of tensile to shear cracks. Between 5 and 8 MPa, the number of micro-cracks increases almost exponentially with stress, while the ratio of tensile to shear cracks decreases from 3 to 2. Comparing Figs 19–21, it is evident that the number of cracks in CGC under the three types of FRP confinement follows the order: CFRP > BFRP > GFRP. This indicates that CFRP provides the most effective confinement, followed by BFRP, with GFRP offering the least confinement.

In summary, the evolution process of micro-cracks can be roughly divided into the crack initiation stage, the rapid development stage, and the slow development stage. In the crack initiation stage, micro-cracks begin to appear inside the structure, with the growth rate of tensile cracks significantly higher than that of shear cracks. As a result, the ratio of tensile to shear cracks increases rapidly, as shown in the pink area in the figure. In the rapid development stage, the number of micro-cracks inside the structure increases sharply, and the growth rate of shear cracks exceeds that of tensile cracks. This leads to a gradual decrease in the ratio of tensile to shear cracks, although tensile cracks remain dominant, as indicated by the blue section in the figure. In the slow development stage, the development rate of micro-cracks inside the structure slows down, and the growth rates of tensile and shear cracks become nearly equal, with the ratio of tensile to shear cracks remaining almost constant, as shown in the green area in the figure.

## 6 Conclusion

In this study, an FDM-DEM coupled modeling method was employed to establish a 3D numerical model of concrete with different coal gangue replacement rates, confined by two layers of FRP (GFRP, CFRP, and BFRP). Through uniaxial compression simulation tests on coal gangue concrete with different types of FRP confinement, the micro-mechanisms and structural evolution patterns underlying its macroscopic mechanical response were thoroughly analyzed. The main conclusions drawn were as follows:

1. Uniaxial compression tests were conducted on both unconfined specimens and specimens confined with two layers of GFRP. The results indicate that the peak stress of the specimens with two layers of GFRP increased by 59%, 42%, and 50% under coal gangue replacement rate of 0%, 50% and 100%, respectively. Additionally, the peak strains were 8.25, 4.75, and 4.75 times higher than those of the unconfined specimens, respectively. GFRP confinement significantly enhances the peak stress and peak strain of coal gangue concrete, thereby improving its compressive strength and deformation capacity. However, as the coal gangue replacement rate increases, the effectiveness of GFRP confinement diminishes, leading to a reduction in both compressive strength and ductility.
2. The stress-strain curves of coal gangue concrete specimens confined by GFRP, CFRP, and BFRP exhibit a bilinear characteristic. Both compressive strength and elastic modulus decrease as the coal gangue replacement rate increases. Specifically, for CFRP confined specimens, the compressive strength decreased by 40% and 55%, while the elastic modulus decreased by 3.1% and 30% under 50% and 100% replacement rates, respectively. For BFRP confined specimens, the compressive strength decreased by 27% and 43%, while the elastic modulus decreased by 3.2% and 15%, respectively. For GFRP confined specimens, the compressive strength decreased by 21% and 39%, while the elastic modulus decreased by 9.8% and 20%, respectively. Overall, the confinement strength of the three types of FRP rank as follows: CFRP > BFRP > GFRP.
3. The type of FRP confinement has a significant effect on the distribution of contact number and contact force of the specimens. For GFRP and CFRP confined specimens, the maximum values of contact force and contact number initially increase and then decrease as the replacement rate increases. In contrast, BFRP confined specimens exhibit a trend of decreasing initially followed by an increase, reflecting the significant influence of FRP material stiffness and flexibility on the confinement effectiveness and load-bearing capacity of the specimens under different replacement rates. However, the contact number of all specimens consistently display a ‘peanut’ shape distribution, while the contact force exhibits an approximately ‘elliptical’ shape distribution.
4. The crack evolution characteristics of GFRP, CFRP, and BFRP-confined coal gangue concrete were studied during the loading process. It was observed that coal gangue concrete under three types of FRP confinement experiences an initiation phase, a rapid development phase, and a slow growth phase, ultimately forming an ‘X’ shaped crack distribution. The ratio of tensile to shear cracks in GFRP, CFRP, and BFRP confined specimens initially increased followed by a decrease, ultimately stabilizing at 2.5, 1 and 2, respectively. The confinement effectiveness of three types of FRP on coal gangue concrete specimens ranks as follows: CFRP > BFRP > GFRP.

## Supporting information

S1 Fig 10 Comparison of experimental and simulation results of GFRP confined specimens. (XLSX)

S2 Fig 11 Stress-strain curves of FRP confined specimens. (XLSX)

S3 Fig 12 Enhancement of stress and strain for FRP confined specimens. (XLSX)

S4 Fig 13 Compressive strength of FRP confined CGC. (XLSX)

S5 Fig 14 Elastic modulus of FRP confined CGC. (XLSX)

S6 Fig 18 The maximum values of strong contact force and strong contact number for FRP confined CGC. (XLSX)

S7 Fig 19 Crack propagation characteristics in the failure of GFRP confined coal gangue concrete. (XLSX)

S8 Fig 20 Crack propagation characteristics in the failure of CFRP confined coal gangue concrete. (XLSX)

S9 Fig 21 Crack propagation characteristics in the failure of BFRP confined coal gangue concrete. (XLSX)
